# Iohexol plasma clearance in children: validation of multiple formulas and single-point sampling times

**DOI:** 10.1007/s00467-017-3841-y

**Published:** 2017-11-13

**Authors:** Camilla Tøndel, Cathrin Lytomt Salvador, Karl Ove Hufthammer, Bjørn Bolann, Damien Brackman, Anna Bjerre, Einar Svarstad, Atle Brun

**Affiliations:** 1 Department of Pediatrics, Haukeland University Hospital, 5021 Bergen, Norway; 2 Department of Clinical Medicine, University of Bergen, Bergen, Norway; 3 Department of Medical Biochemistry, Oslo University Hospital, Oslo, Norway; 4 Institute of Clinical Medicine, Faculty of Medicine, University of Oslo, Oslo, Norway; 5 Centre for Clinical Research, Haukeland University Hospital, Bergen, Norway; 6 Laboratory for Clinical Biochemistry, Haukeland University Hospital, Bergen, Norway; 7 Department of Clinical Science, University of Bergen, Bergen, Norway; 8 Department of Pediatrics, Oslo University Hospital, Oslo, Norway; 9 Department of Medicine, Haukeland University Hospital, Bergen, Norway

**Keywords:** Glomerular filtration rate, Children, Chronic kidney disease, Renal function

## Abstract

**Background:**

The non-ionic agent iohexol is increasingly used as the marker of choice for glomerular filtration rate (GFR) measurement. Estimates of GFR in children have low accuracy and limiting the number of blood-draws in this patient population is especially relevant. We have performed a study to evaluate different formulas for calculating measured GFR based on plasma iohexol clearance with blood sampling at only one time point (GFR1p) and to determine the optimal sampling time point.

**Methods:**

Ninety-six children with chronic kidney disease (CKD) stage 1–5 (median age 9.2 years; range 3 months to 17.5 years) were examined in a cross-sectional study using iohexol clearance and blood sampling at seven time points within 5 h (GFR7p) as the reference method. Median GFR7p was 66 (range 6–153) mL/min/1.73 m^2^. The performances of six different single time-point formulas (Fleming, Ham and Piepsz, Groth and Aasted, Stake, Jacobsson- and Jacobsson-modified) were validated against the reference. The two-point GFR (GFR2p) was calculated according to the Jødal and Brøchner–Mortensen formula.

**Results:**

The GFR1p calculated according to Fleming with sampling at 3 h (GFR1p_3h_-Fleming) had the best overall performance, with 82% of measures within 10% of the reference value (P10). In children with a GFR ≥ 30 mL/min/1.73 m^2^ (*n* = 78), the GFR1p_3h_-Fleming had a P10 of 92.3%, which is not significantly different (*p* = 0.29) from that of GFR2p (P10 = 96.2%). Considerable differences within and between the different formulas were found for different CKD stages and different time points for blood sampling.

**Conclusions:**

For determination of mGFR in children with CKD and an assumed GFR of ≥ 30 mL/min/1.73 m^2^ we recommend GFR1p_3h_-Fleming as the preferred single-point method as an alternative to GFR2p. For children with a GFR < 30 mL/min/1.73 m^2^, we recommend the slope-GFR with at least two blood samples.

**Clinical Trial Registration**: ClinicalTrials.gov, Identifier NCT01092260, https://clinicaltrials.gov/ct2/show/NCT01092260?term=tondel&rank=2

## Introduction

The low accuracy of formulas for estimating glomerular filtration rate (GFR) in children has long been a major challenge, with studies showing that less than 50% of the GFR levels estimated (eGFR) using formulas based on serum cystatin C, creatinine and/or urea are within ± 10% of the gold standard GFR measurement [1]. In pediatric nephrology care, more accurate determinations of kidney function are therefore needed with a feasible measured GFR (mGFR) methodology based on the plasma clearance of an exogenous GFR marker. Since the 1980s, GFR has been increasingly measured using the non-ionic agent iohexol [2–7]. To avoid extended examinations with multiple blood samples for measuring GFR, many centers have chosen to use the one-pool slope-intercept technique with a minimum of two blood samples [8–11]. Numerous single-point GFR (GFR1p) methods have been developed, and especially in pediatric care, it is clearly beneficial to reduce the number of blood draws from two or three to a single sample, provided an adequate level of accuracy can be preserved [11–14]. However, current guidelines from the British Nuclear Medicine Society (BNMS) do not endorse the routine use of a GFR1p method and recommend a one-pool slope-intercept technique requiring at least two samples [11]. The GFR1p methodology was introduced in adult patients by Fisher and colleagues in 1975 based on ^51^Cr-EDTA clearance [15], and an improved concept was described by Groth and colleagues in 1981 [16]. In 1983, Jacobsson published a formula for GFR1p which takes into account different distribution volumes and different sampling time points in adults based on ^99^TC^m^-DTPA clearance [12]. The Jacobsson formula has been widely used for GFR1p with different markers. Confusingly, modified versions of the Jacobsson formula have also been used but *reported* as being Jacobsson’s original formula [14, 17–19]. Here we report results for Jacobsson’s original adult single-point formula [12] and for the modified, non-iterative formula [14, 17], which does *not* include Jacobsson’s correction for non-uniform distribution. Groth and Aasted published the first pediatric GFR1p formula in 1984 in which they used ^51^Cr-EDTA clearance with a sampling point at 2 h [20]. In 1991, Ham&Piepsz published a new formula for GFR1p in children, also with sampling at 2 h and based on ^51^Cr-EDTA clearance. A modification of the Jacobsson formula for pediatric use was published the same year by Stake and colleagues; these authors recommended a sampling point at 3 h based on ^99^TC^m^-DTPA clearance [3, 21]. In 2005, Fleming and colleagues described a new formula for GFR1p which they developed from a cohort of 100 children and 225 adults; this formula provided GFR values consistent with those obtained by the slope-intercept technique [22]. Although the Fleming formula first and foremost was suggested as a quality control method for the slope-intercept technique [22], a recent study [19] reports results arguing for the GFR1p-Fleming as a potential stand-alone formula for pediatric nephrology care.

The aims of our study were to: (1) assess the accuracy of the different formulas for GFR1p determination by comparison with the reference iohexol seven-point plasma clearance measurements (GFR7p) and (2) determine the optimal single time point for blood sampling for GFR1p within a feasible time frame (i.e. blood sampling not later than 5 h after injection).

## Patients and methods

### Patients

Ninety-six children with chronic kidney disease (CKD) were recruited in a cross-sectional study (ClinicalTrials.gov Identifier NCT01092260) which has evaluated the two-point methodology [23]: 54 children at Haukeland University Hospital, Bergen, Norway, and 42 children at Oslo University Hospital, Oslo, Norway. The median age of the included children (55 males, 41 females), was 9.2 years (range 3 months to 17.5 years), the median weight was 28.3 (range 6.6–84.6) kg and the median height was 134 (range 59–177) cm. Median reference GFR based on seven blood sample time points (GFR7p) was 66 (range 6–153) mL/min/1.73 m^2^. The individual GFR measurements were divided between the different GFR stages, namely, from 28, 27, 23, and 18 patients in CKD stage 1, 2, 3 and 4–5, respectively.

### Methods

Iohexol was administered as Omnipaque®300 mg I/mL (647 mg iohexol/mL; GE Healthcare Technologies Norway AS, Oslo, Norway) in a dose adapted to body weight. Blood samples were drawn at 10, 30 or 60, 120, 180, 210, 240 and 300 min after injection. Additional details are provided in an earlier study published on the same cohort with a focus on two-point methodology [23].

### Calculations and statistics

The GFR7p was calculated according to Sapirstein, as described by Schwartz et al. [3, 24] (Tables 1, 2). A two-compartment model was fitted using linear regression of the log concentration values. For three patients the two-compartment slope-intercept method could not be used due to negative values after the slow component of the curve was removed; for these three patients, we fitted the two-compartment model directly using non-linear least squares. GFR was normalized to 1.73 m^2^ body surface area (BSA) by the ratio 1.73/BSA, using the formula of Haycock et al. [25]. The GFR1p was calculated with six different formulas: the Fleming formula [22], the Ham and Piepsz formula (Ham&Piepsz; [26], the Stake formula [13, 21], the Groth and Aasted formula (Groth&Aasted; [20]), the Jacobsson formula [12] and a modification of Jacobsson’s formula (GFR1p-Jacobsson-mod.) [14, 17] which is based on performing only the first step in Jacobsson’s three-step GFR calculation. Tables 1 and 2 show the formulas used in the calculation of the GFR values, along with numerical examples. One patient had an obviously incorrect value measured for the 3.5-h sample, and this value was therefore removed before the analyses; otherwise the data were complete, with no missing values.

**Table 1:** Methodology of glomerular filtration rate calculations

Name of method/reference^a^	Formula^b^
Reference GFR (GFR7p)	
Two-compartment model	*C*(*t*) = *Ae* ^−*αt*^ + *Be* ^−*βt*^
Absolute GFR7p (mL/min)^b^	*Cl* = I/(*A*/*α* + *B*/*β*)
BSA-normalized GFR7p (mL/min/1.7 3 m^2^)	*Cl*_BSA_ = *Cl* × 1.73/*BSA*
Single-point GFR (GFR1p)	
GFR1p-Fleming [11]	${V}_{app}(t)=\frac{I}{C(t)}\times 1.73/ BSA$
	*A* = − 11297 − 4883 ∙ *BSA* − 41.94 ∙ *t*
	*B* = 5862 + 1282 ∙ *BSA* + 15.5 ∙ *t*
	$$ {Cl}_{BSA}^{\prime }=\frac{A+B\bullet \ln \frac{V_{app(t)}}{1000}}{t} $$
	$$ {Cl}_{BSA}=\max \left({Cl}_{BSA}^{\prime },0\right) $$
GFR1p-Ham&Piepz [26]	*C*_120_ = *C*(*t*) · exp[0.008 · (t − 120)]
	*V*_120_ = *I*/*C*_120_
	*Cl* = 2.602 · *V*_120_/1000 − 0.273
	*Cl*_*BSA*_ = *Cl* · 1.73/*BSA*
GFR1p-Groth&Aasted [16]	*A* = − 72.295 ∙ ln(*t*) + 425.41
	*B* = − 553.124 ∙ ln(*t*) + 3236.76
	$$ x=\ln \left(\frac{I}{C(t)\bullet BSA\bullet {10}^7}\right) $$
	*Cl*_*BSA*_ = *A* · *x* + *B*
GFR1p-Stake [13, 21]	${V}^{\prime }=231\cdotp \mathsf{weight}+1215$
	$$ {Cl}^{\prime }=\ln \left(\frac{I}{V^{\prime}\bullet C(t)}\right)/\left(\frac{I}{V^{\prime}\bullet C(t)}+0.0016\right) $$
	$$ {Cl}_{BSA}^{\prime }={Cl}^{\prime}\cdotp 1.73/ BSA $$
	*Cl*_*BSA*_ = 180 − 14.1 √ [133 − min(*Cl*′_*BSA*_, 133)]
GFR1p-Jacobsson [12]	$V=246\cdotp \mathsf{weight}$ ${Cl}_v=\ln \left(\frac{I}{V\bullet C(t)}\right)/\left(\frac{I}{V\bullet C(t)}+0.0016\right)$ *m* = 0.991 − 0.00122 × *Cl*_*v*_ *V* ^′^ = *V*/*m*
	$$ Cl=\ln \left(\frac{I}{V^{\prime}\bullet C(t)}\right)/\left(\frac{I}{V^{\prime}\bullet C(t)}+0.0016\right) $$
	$$ {Cl}_{BSA}=\mathrm{Cl}\cdotp 1.73/\mathrm{BSA} $$
GFR1p-Jacobsson-mod. [17]	$V=246\cdotp \mathsf{weight}$
	$$ Cl=\ln \left(\frac{I}{V\bullet C(t)}\right)/\left(\frac{I}{V\bullet C(t)}+0.0016\right) $$
	$$ {Cl}_{BSA}=\mathrm{Cl}\cdotp 1.73/\mathrm{BSA} $$

See Table 2 for additional formulas and an example

^a^GFR, Glomerular filtration rate; GFR7p, reference GFR based on seven blood sample time points; GFR1p, GFR value based on blood-draw at one time point

^b^ *I*, the dose of iohexol in mg; *C(t)*, the concentration in mg/mL at *t* min after injection; BSA, body surface area in m^2^, calculated according to Haycock [25]; *V* and *V*´, estimated volume of distribution; *Cl*, unadjusted GFR; *Cl*_BSA_, BSA-adjusted GFR estimate

^c^See Patients and methods sections for additional information on calculation of the GFR7p value

**Table 2:** Example data, with additional information on calculations

Examples	Value	Units	Calculation/comment
Product			
Omnipaque	300	mg I/ml	
Product density	1.345	g/ml	Product density at room temperature.
Iohexol density	647	mg/mL	
Injected dose			
Omnipaque, weight	2.8	g	
Omnipaque, volume	2.08	mL	2.8 g/1.345 g/mL
Iohexol, weight	1346.9	mg	2.08 mL × 647 mg/mL
Example patient			
Sample time	180	min	3 h × 60 min/h
Concentration	0.100	mg/mL	100 μg/mL
Body weight	13	kg	
Body height	90	cm	
BSA	0.574	m^2^	$0.024265\times {\mathsf{height}}^{0.3964}\times {\mathsf{weight}}^{0.3964}$ =0.024265 × 90^0.3964^ × 13^0.5378^
GFR1p values (BSA-adjusted)			
GFR1p-Fleming	72.9	mL/min/1.73 m^2^	See Table 1
GFR1p-Ham&Piepz	64.6	mL/min/1.73 m^2^	See Table 1
GFR1p-Groth&Aasted	61.8	mL/min/1.73 m^2^	See Table 1
GFR1p-Stake	76.5	mL/min/1.73 m^2^	See Table 1
GFR1p-Jacobsson	75.7	mL/min/1.73 m^2^	See Table 1
GFR1p-Jacobsson-mod.	74.9	mL/min/1.73 m^2^	See Table 1
GFR7p calculations			
Measured concentrations at all time points:			
Time point	Time (min)	*C*(*t*) (mg/mL)	*C* ^∗^(*t*) (mg/mL)
1	10	0.464	0.169
2	30	0.343	0.082
3	120	0.156	–
4	180	0.100	–
5	210	0.084	–
6	240	0.072	–
7	300	0.051	–

Two-compartment model: *C*(*t*) = *Ae* ^−*αt*^ + *Be* ^−*βt*^ = fast part + slow part

Regression of ln(*C*(*t*)) on *t* for the slow part (time point 3–7):

(Intercept) ln(*B*) = − 1.16 ⇒ *B* = 0.31

(Slope) −*β* = − 0.0061 ⇒ *β* = 0.0061

*C* ^∗^(*t*) is the concentration after removing the slow part of the curve:

*C* ^∗^(*t*) = *C*(*t*) − *Be* ^−*βt*^ = *C*(*t*) − 0.31*e* ^−0.0061*t*^

Regression of ln(*C* ^∗^(*t*)) on *t* (time point 1–2):

Intercept: ln(*A*) = − 1.42 ⇒ *A* = 0.24

Slope: −*α* = − 0.036 ⇒ *α* = 0.036

AUC for fast part: $\frac{A}{\alpha }=\frac{A}{\alpha }=6.7$

AUC for slow part: $\frac{B}{\beta }=\frac{B}{\beta }=51.1$

Total AUC = 51.1 + 6.7 = 57.8

Unadjusted GFR**: $$ Cl=\frac{I}{AUC}=\frac{I}{AUC}=23.3 $$

Glomerular filtration rate (GFR) adjusted for body surface area (BSA): *Cl*_*BSA*_ = *GFR* · 1.73/*BSA* = 70.3. Note that the final calculations are based on more decimals than are shown in the intermediate calculations

**Fig. 1:**
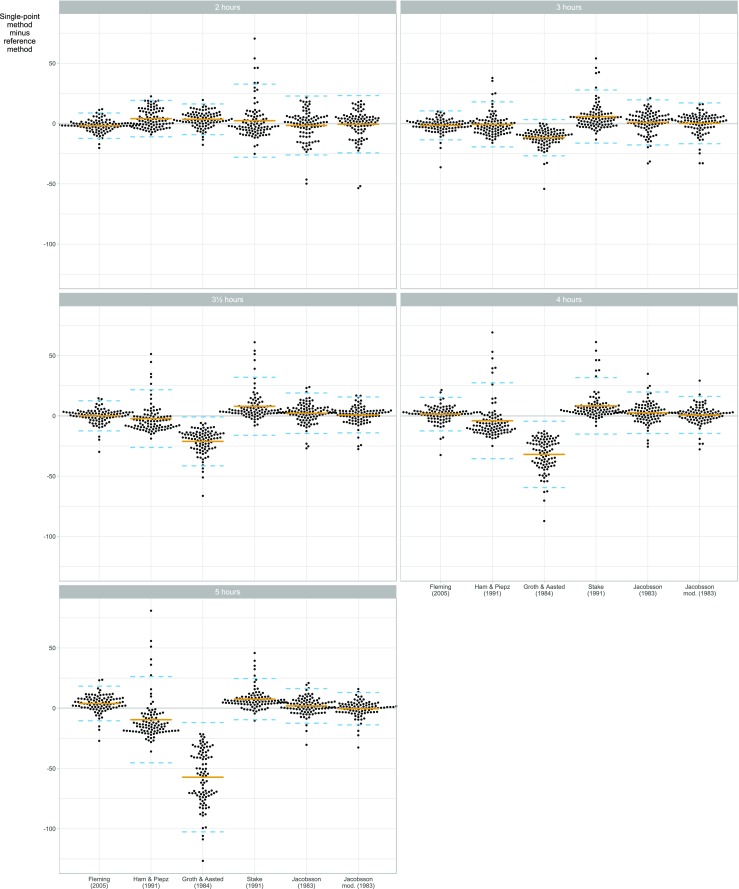
Plot of estimation error versus the estimation method for glomerular filtration rate (GFR) calculated by six single-sample formulas [12, 13, 17, 20, 22, 26], stratified by sampling time point (*n* = 96 children). The *y*-axis shows the difference between the single-point GFR and a reference GFR based on seven sampling time points. Each point corresponds to a combination of patient, estimation method and sample time. The solid horizontal line is the bias, i.e. the mean difference between the single-point GFR estimate and the reference GFR. The dashed lines are limits of agreement, i.e. bias ± two standard deviations of the differences. The figure can be used to compare different methods *within* each sampling time point

**Fig. 2:**
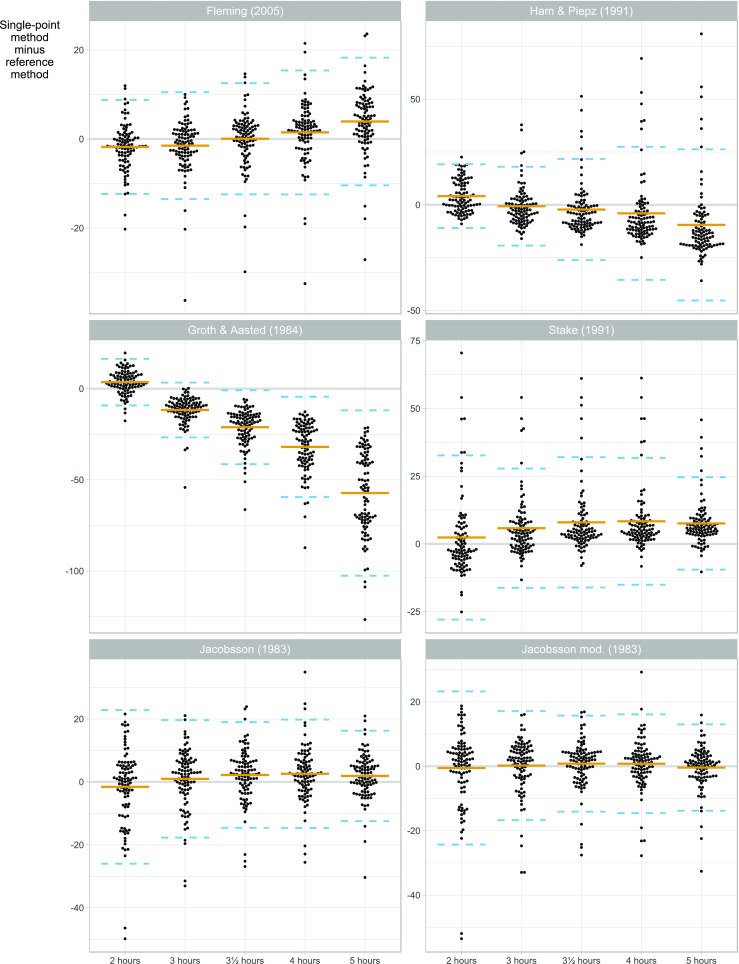
Plot of estimation error versus time point for glomerular filtration rate (GFR) calculated at five time points, stratified by estimation method (*n* = 96 children). The *y*-axis shows the difference between the single-point GFR [12, 13, 17, 20, 22, 26] and a reference GFR based on seven sampling time points. Each point corresponds to a combination of patient, estimation method and sample time. The solid horizontal line is the bias, i.e. the mean difference between the single-point GFR and the reference GFR. The dashed lines are limits of agreement, i.e. bias ± two standard deviations of the differences. For each estimation method, the figure can be used to compare the performance of the single-point GFR estimates at different sampling time-points

**Fig. 3:**
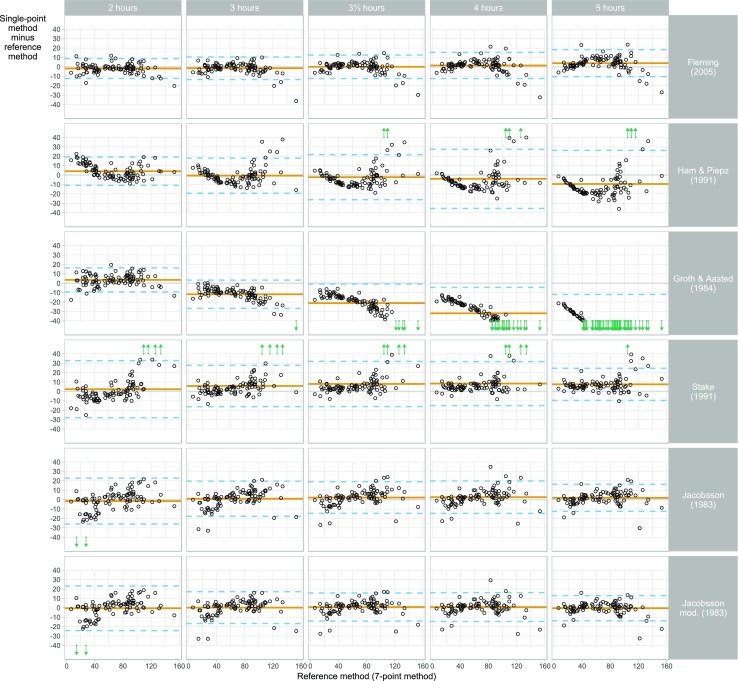
Plot of estimation error versus reference glomerular filtration rate (GFR) for GFR calculated by six single-sample formulas [12, 13, 17, 20, 22, 26] and at five sampling time points (*n* = 96 children). The *y*-axis shows the difference between the single-point GFR estimate and a reference GFR based on seven sampling time points. The *x*-axis shows the reference GFR. Each point corresponds to a combination of patient, determination method and sample time. The solid horizontal line is the bias, i.e. the mean difference between the single-point GFR and the reference GFR. The dashed lines are limits of agreement, i.e. bias ± two standard deviations of the differences. Large determination errors, i.e. errors outside the displayed range, are indicated by arrows. The figure can be used to examine patterns in how the estimation errors of the different estimation methods vary with GFR for each method and sampling time

To compare the GFR1p methods and the reference method, we calculated the difference between the various GFR1p and the reference GFR for each patient, along with estimated bias (mean difference) and limits of agreement (bias ± twice the standard deviation of the differences). The data are presented as difference plots comparing: (1) different methods within a single sampling time point (Fig. 1), (2) different sampling time points for each method (Fig. 2), and (3) the bias for different GFR values for each method (Fig. 3). We also present the corresponding numerical estimates for the time points recommended in the original publications (Table 3) and for various subgroups (Table 5) according to age (< 10 years and ≥ 10 years), BSA group (< 1.0 m^2^ and < 1.45 m^2^) and stage of CKD (< 30 mL/min/1.73 m^2^, 30 to < 60 mL/min/1.73 m^2^, 60 to < 90 mL/min/1.73m^2^, ≥ 90 mL/min/1.73m^2^).

**Table 3:** Effect of different formulas at their recommended time points

Formula	Mean bias (GFR1p − GFR7p)				Proportion of measures within *x*% of reference method (95% CI)^a^								*P* value^a^
	Time (h)	*r*	Bias	Limits of agreement	P5		P10		P15		P20		
					% of measures	95% CI	% of measures	95% CI	% of measures	95% CI	% of measures	95% CI	
GFR1p-Fleming	2	0.99	− 1.8	− 12.3 to 8.8	54	44–64	79	70–86	86	78–92	90	82–94	0.42
	3	0.98	− 1.5	− 13.5 to 10.5	56	46–66	82	73–89	89	81–93	91	83–95	N/A
	3.5	0.98	0.1	− 12.4 to 12.5	44	35–54	81	72–88	87	79–93	89	82–94	0.51
	4	0.98	1.5	− 12.4 to 15.4	41	31–51	75	65–83	90	82–94	92	84–96	0.10
GFR1p-Ham&Piepz	2	0.98	4.1	− 11.0 to 19.2	42	32–52	64	54–72	73	63–81	76	67–83	< 0.001
GFR1p-Groth&Aasted	2	0.98	3.6	− 9.2 to 16.3	35	27–45	65	55–73	75	65–83	81	72–88	< 0.001
GFR1p-Stake	3	0.97	5.8	− 16.2 to 27.8	33	25–3	66	56–74	78	69–85	82	73–89)	0.002
GFR1p-Jacobsson	2	0.97	− 1.6	− 26.0 to 22.8	32	24–42	58	48–68	69	59–77	78	69–85	< .001
	3	0.98	1.0	− 17.7 to 19.7	34	26–44	58	48–68	74	64–82	81	72–88	< .001
	3.5	0.98	2.2	− 14.6 to 19.0	27	19–37	64	54–73	75	65–82	82	73–89	< .001
	4	0.98	2.6	− 14.6 to 19.8	32	24–42	65	55–73	76	67–83	88	79–93	< .001
	5	0.98	1.9	− 12.5 to 16.3	43	33–53	72	62–80	85	77–91	91	83–95	0.04
GFR1p-Jacobsson-mod.	2	0.97	− 0.5	− 24.3 to 23.2	33	25–43	61	51–71	70	60–78	78	69–85	< 0.001
	3	0.98	0.2	− 16.7 to 17.1	35	27–45	69	59–77	75	65–83	84	76–90	0.003
	3.5	0.98	0.8	− 14.1 to 15.7	41	32–51	65	55–74	80	71–87	88	80–93	< 0.001
	4	0.98	0.8	− 14.5 to 16.1	42	32–52	71	61–79	85	77–91	91	83–95	0.02
	5	0.98	− 0.4	− 13.8 to 13.0	50	40–60	74	64–82	90	82–94	94	87–97	0.08
GFR2p-JBM	2 and 5	0.99	− 1.7	− 9.4 to 6.1	73	63–81	97	97–99	100	96–100	100	96–100	< 0.001

Evaluation of optimal time for blood sampling was investigated using five different sampling time points after iohexol injection, namely 2, 3, 3.5, 4 and 5 h. glomerular filtration rate (GFR) (mL/min/1.73m^2^) was estimated by one-point methods at time points recommended in the original publications (*n* = 96 for all time points except for 3.5 h, where *n* = 95) and by the reference method (GFR7p). Mean bias, 95% limits of agreement and correlation (*r*) with reference method are shown calculated. For comparison the two-point method of Jødal Brøchner Mortensen (GFR2p-JBM) was added

N/A, Not applicable

^a^Estimated accuracy is shown as P5, P10, P15 and P20, namely, the percentage of patients within ± 5, 10, 15 and 20% of the reference method, respectively, along with 95% confidence intervals (CI)

^b^Comparison with Fleming P10 at 3 h

**Fig. 4:**
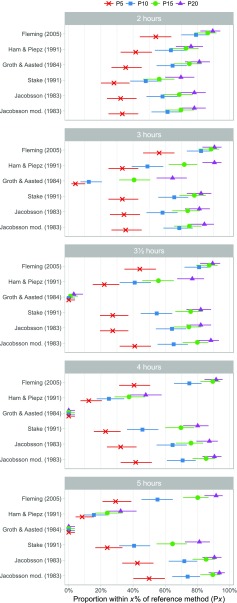
Percentage plot showing the determination accuracy of six single-sample determination methods [12, 13, 17, 20, 22, 26] at five sampling time points (*n* = 96 patients/children). Each symbol, labeled P*x* (P5, P10, P15 and P20), shows the calculated proportion of single-sample glomerular filtration rate (GFR) within *x*% of the reference method. The horizontal lines show the corresponding 95% confidence intervals

To further quantify the performance of the GFR1p-methods, we calculated the number of GFR values that were within 5%, 10%, 15% or 20% of the reference method for each formula, labeled as P5, P10, P15 and P20, respectively, along with confidence intervals based on the recommended Wilson method [27] (Tables 3, 4, and 5; Fig. 4). Differences between methods and between time points for these ‘P*x*’ values (*x* = 5, 10, 15 or 20) were evaluated using the McNemar test with mid-*P* correction.

**Table 4:** Percentage of GFR1p measures within 10% of reference stratified by GFR-levels

Formula	Time (h)	Proportion of measures within 10% (P10) of reference method (95% CI)											
		CKD 1		CKD 2		CDK 3		CKD 1–3		CKD 4–5		CKD 1–5	
		% of measures	95% CI	% of measures	95% CI	% of measures	95% CI	% of measures	95% CI	% of measures	95% CI	% of measures	95% CI
GFR1p-Fleming	2	97	83– 99	88	71–96	78	58–90	88	80– 94	39	20–61	79	70–86
	3	90	74–96	96	81–99	91	73–98	92	84–96	39	20–61	82	73–89
	3.5	83	65–92	96	81–99	91	73–98	90	81–95	41	22–64	81	72–88
	4	79	62–90	85	66–94	78	58–90	81	71–88	50	29–71	75	65–83
	5	69	51–83	46	29–65	39	22–59	53	42 –63	67	44–84	55	45–65
GFR1p-Ham&Piepz	2	79	62–90	96	81–99	57	37–74	78	68–86	0	0–18	64	54–72
	3	62	44–77	54	35–71	39	22–59	53	42–63	33	16–56	49	39–59
	3.5	66	47–80	35	19–54	13	5–32	40	30–51	47	26–69	41	32–51
	4	41	26–59	35	19–54	0	0–14	27	18–38	17	6–39	25	17–35
	5	34	20–53	19	9–38	0	0–14	19	12–29	0	0–18	16	10–24
GFR1p-Groth&Aasted	2	79	62–90	81	62–91	57	37–74	73	62–82	28	12–51	65	55–73
	3	14	5–31	12	4–29	4	1–21	10	5–19	22	9–45	12	7–21
	3.5	0	0–2	0	0–13	0	0–14	0	0–5	0	0–18	0	0–4
	4	0	0–12	0	0–13	0	0–14	0	0–5	0	0–18	0	0–4
	5	0	0–12	0	0–13	0	0–14	0	0–5	0	0–18	0	0–4
GFR1p-Stake	2	48	31–66	81	62–91	35	19–55	55	44–66	17	6–39	48	38–58
	3	45	28–62	85	66–94	91	73–98	72	61–81	39	20–61	66	56–74
	3.5	45	28–62	77	58–89	65	45–81	62	50–72	24	10–47	55	45–64
	4	55	38–72	58	39–74	52	33–71	55	44–66	6	1–26	46	36–56
	5	55	38–72	62	43–78	22	10–42	47	37–58	11	3–33	41	31–51
GFR1p-Jacobsson	2	66	47–80	77	58–89	48	29–67	64	53–74	33	16–56	58	48–68
	3	62	44–77	73	54–86	61	41–78	65	54–75	28	12–51	58	48–68
	3.5	76	58–88	77	58–89	61	41–78	72	61–81	29	13–53	64	54–73
	4	79	62–90	65	46–81	70	49–84	72	61–81	33	16–56	65	55–73
	5	86	69–95	77	58–89	70	49–84	78	68–86	44	25–66	72	62–80
GFR1p-Jacobsson-mod	2	69	51–83	81	62–91	52	33–71	68	57–77	33	16–56	61	51–71
	3	83	65–92	85	66–94	65	45–81	78	68–86	28	12–51	69	59–77
	3.5	79	62–90	77	58–89	61	41–78	73	62–82	29	13–53	65	55–74
	4	79	62–90	81	62–91	83	63–93	81	71–88	28	12–51	71	61–79
	5	72	54–85	96	81–99	74	54–87	81	71–88	44	25–66	74	64–82
GFR2p-Jødal-Brøchner-Mortensen	2 and 5	90	74–96	100	87–100	100	86–100	96	89–99	100	82–100	97	91–99

*CKD* Chronic kidney disease; *GFR* glomerular filtration rate; *CI* confidence interval

For comparison, subanalyses for data on the best available two-point methodology (GFR2p) (Jødal and Brøchner-Mortensen [23, 28, 29]) at 2 and 5 h (i.e. GFR2p-JBM) are also included in Tables 3, 4, and 5.

**Table 5:** Subgroup analysis

Patient group	Formula	Time (h)	*n*	*r*	Mean bias (GFR1p − GFR7p)		Percentage of measures within *x*% of reference method (P*x*)^a^				*P* value^b^
					Bias	Limits of agreement	P5	P10	P15	P20	
Age < 10 years	GFR1p-Fleming	3	52	0.99	− 0.5	− 9.7 to 8.8	65	83	88	92	N/A
	GFR1p-Ham&Piepz	2	52	0.97	2.3	− 13.3 to 17.8	48	73	83	87	0.15
	GFR1p-Groth&Aasted	2	52	0.99	2.0	− 10.7 to 14.7	40	69	81	87	0.02
	GFR1p-Stake	3	52	0.97	7.3	− 14.1 to 28.6	29	62	75	79	0.008
	GFR1p-Jacobsson	5	52	0.98	2.0	− 11.1 to 15.1	52	79	90	92	0.55
	GFR1p-Jacobsson-mod.	5	52	0.98	0.1	− 12.8 to 13.0	58	81	90	94	0.77
	GFR2p-JBM	2 and 5	52	0.99	− 2.0	− 10.2 to 6.3	60	96	100	100	0.02
Age ≥ 10 years	GFR1p-Fleming	3	44	0.98	− 2.7	− 17.1 to 11.6	45	82	89	89	N/A
	GFR1p-Ham&Piepz	2	44	0.99	6.3	− 7.1 to 19.6	34	52	61	64	0.001
	GFR1p-Groth&Aasted	2	44	0.99	5.4	− 6.6 to 17.3	30	59	68	75	0.02
	GFR1p-Stake	3	44	0.98	4.1	− 18.5 to 26.6	39	70	82	86	0.11
	GFR1p-Jacobsson	5	44	0.98	1.8	− 14.1 to 17.7	32	64	80	89	0.04
	GFR1p-Jacobsson-mod.	5	44	0.98	− 1.0	− 15.0 to 13.0	41	66	89	93	0.02
	GFR2p-JBM	2 and 5	44	1.0	− 1.4	− 0.5 to 5.8	89	98	100	100	0.02
BSA < 1.0 m^2^	GFR1p-Fleming	3	47	0.99	0.0	− 9.5 to 9.6	68	81	87	91	N/A
	GFR1p-Ham&Piepz	2	47	0.97	2.9	− 13.0 to 18.7	43	68	79	83	0.09
	GFR1p-Groth and Aasted	2	47	0.99	2.1	− 11.3 to 15.5	45	64	79	85	0.01
	GFR1p-Stake	3	47	0.98	7.6	− 12.1 to 27.3	23	57	72	77	0.008
	GFR1p-Jacobsson	5	47	0.97	1.5	− 12.4 to 15.4	57	83	89	89	0.73
	GFR1p-Jacobsson-mod.	5	47	0.98	− 0.2	− 13.6 to 13.3	60	83	91	94	0.75
	GFR2p-JBM	2 and 5	47	0.99	− 1.9	− 10.1 to 6.3	60	96	100	100	0.02
BSA < 1.45 m^2^	GFR1p-Fleming	3	77	0.99	− 0.4	− 9.6 to 8.9	60	84	90	92	N/A
	GFR1p-Ham&Piepz	2	77	0.97	3.4	− 11.7 to 18.4	42	65	77	81	< 0.001
	GFR1p-Groth&Aasted	2	77	0.98	3.1	− 9.5 to 15.6	39	68	78	86	0.001
	GFR1p-Stake	3	77	0.97	7.2	− 15.6 to 30.0	30	64	75	81	< 0.001
	GFR1p-Jacobsson	5	77	0.98	2.6	− 10.8 to 15.9	48	75	87	92	0.10
	GFR1p-Jacobsson-mod.	5	77	0.98	0.6	− 11.5 to 12.6	55	77	91	96	0.17
	GFR2p-JBM	2 and 5	77	0.99	− 1.7	− 9.7 to 6.3	69	96	100	100	0.01
CKD stage 1	GFR1p-Fleming	3	29	0.83	− 4.1	− 21.9 to 13.8	59	90	93	97	N/A
	GFR1p-Ham&Piepz	2	29	0.93	2.6	− 12.6 to 17.8	59	79	97	100	0.34
	GFR1p-Groth&Aasted	2	29	0.91	4.3	− 8.7 to 17.3	48	79	97	100	0.34
	GFR1p-Stake	3	29	0.82	14.9	− 15.4 to 45.1	21	45	66	72	0.001
	GFR1p-Jacobsson	5	29	0.82	2.6	− 17.0 to 22.3	45	86	86	93	0.69
	GFR1p-Jacobsson-mod.	5	29	0.81	− 2.9	− 21.5 to 15.7	55	72	97	97	0.03
	GFR2p-JBM	2 and 5	29	0.92	− 1.7	− 15.3 to 9.1	59	90	100	100	0.81
CKD stage 2	GFR1p-Fleming	3	26	0.93	0.4	− 6.8 to 7.7	73	96	100	100	N/A
	GFR1p-Ham&Piepz	2	26	0.91	− 0.7	− 9.5 to 8.0	62	96	96	96	0.75
	GFR1p-Groth&Aasted	2	26	0.91	5.5	− 3.5 to 14.5	42	81	92	96	0.06
	GFR1p-Stake	3	26	0.89	3.9	− 7.1 to 15.0	38	85	92	96	0.13
	GFR1p-Jacobsson	5	26	0.93	4.6	− 4.3 to 13.5	42	77	96	100	0.03
	GFR1p-Jacobsson-mod.	5	26	0.94	2.1	− 5.5 to 9.7	58	96	96	100	0.75
	GFR2p-JBM	2 and 5	26	0.96	− 2.0	− 7.6 to 3.7	69	100	100	100	0.50
CKD stage 3	GFR1p-Fleming	3	23	0.97	0.0	− 4.8 to 4.9	61	91	100	100	N/A
	GFR1p-Ham&Piepz	2	23	0.77	3.7	− 6.2 to 13.6	30	57	70	78	0.01
	GFR1p-Groth&Aasted	2	23	0.78	1.9	− 9.5 to 13.3	35	57	65	74	0.004
	GFR1p-Stake	3	23	0.96	0.6	− 5.0 to 6.2	48	91	100	100	1.00
	GFR1p-Jacobsson	5	23	0.90	2.1	− 7.0 to 11.3	43	70	96	96	0.03
	GFR1p-Jacobsson-mod.	5	23	0.90	2.0	− 6.3 to 10.3	48	74	96	96	0.06
	GFR2p-JBM	2 and 5	23	0.98	− 0.7	− 3.7 to 2.4	87	100	100	100	0.25
CKD stages 4–5	GFR1p-Fleming	3	18	0.83	− 2.0	− 11.7 to 7.6	22	39	50	56	N/A
	GFR1p-Ham&Piepz	2	18	0.75	14.0	5.3–22.8	0	0	6	6	0.008
	GFR1p-Groth&Aasted	2	18	0.77	1.7	−15.3 to 18.7	6	28	28	39	0.45
	GFR1p-Stake	3	18	0.75	0.6	− 11.4 to 12.6	28	39	50	56	0.84
	GFR1p-Jacobsson	5	18	0.73	− 3.4	− 14.7 to 7.8	39	44	56	67	0.75
	GFR1p-Jacobsson-mod.	5	18	0.75	− 2.9	− 13.9 to 8.0	33	44	61	78	0.75
	GFR2p-JBM	2 and 5h	18	1.00	− 0.3	1.2–1.0	83	100	100	100	< 0.001

Evaluation of bias and accuracy for blood sampling for various patient groups and time points after iohexol injection. GFR (mL/min/1.73m ^2^) was determined by one-point methods and by the reference method (GFR7p). Mean bias, 95% limits of agreement and correlation (*r*) with reference method is shown calculated

*GFR* glomerular filtration rate; *CKD* chronic kidney disease; *BSA* body surface area

^a^Estimated accuracy is shown as P5, P10, P15 and P20, the percentage of patients within ±5%, 10%, 15% and 20% of the reference. For comparison, the two-point method of Jødal Brøchner Mortensen (GFR2p-JBM) was added

^b^Comparison with Fleming P10 at 3 h

We used R version 3.4.0 for Windows for all statistical analyses and figures [30]. Statistical significance is defined as *P* values ≤ 0.05, using two-sided tests, not adjusted for multiple comparisons.

## Results

The performances of six different formulas for GFR1p determination [12, 13, 20, 22, 26] compared to the reference method are shown in Tables 3, 4, and 5 and Figs. 1, 2, 3, and 4. The results of different time points of blood sampling in Table 3 are limited to the recommended time points given in the respective original publications. The formula of Fleming with sampling at 3 h (GFR1p_3h_-Fleming) showed the best performance, with 82% of the GFR values falling within 10% of the reference method (P10). For the samplings at 2, 3, and 3.5 hours, the results with the Fleming formula were also significantly better than all the other tested formulas for P10 (Table 3). A comparison between the performances of all tested GFR1p formulas and slope-intercept methodology revealed that the GFR2p-JBM methodology was significantly better than all GFR1p formulas in the entire cohort of children with CKD 1–5 (Tables 3, 4).

With respect to the effect of sampling time on the performance, the Fleming formula gave results for sampling at 2, 3.5 and 4 h (i.e. time frame recommended by Fleming) which were not significantly different from the results at 3 h (Table 3; Figs. 2, 4). However, when blood was drawn at 5 h (i.e. outside the time frame recommended by Fleming), this formula showed a significantly lower performance, with a P10 of 55% (*P* < 0.01) (Table 4; Figs. 2, 4). When sampling at 4 h, the Fleming and Jacobsson-mod. formulas performed significantly better (P10 was 75 and 71%, respectively) than the formulas of Ham & Piepsz, Groth & Aasted and Stake (Figs. 1, 4). For blood sampling at 5 h, the two Jacobsson formulas had the best performance, with a P10 of 74 and 72%, respectively, which was significantly better (*P* < 0.01) than the performance of all other tested formulas at 5 h (Figs. 1, 4; Tables 3, 4).

All GFR1p formulas studied showed large bias when blood was drawn outside the time frames originally described for the respective formulas (Fig. 1). Nevertheless, the formulas of Fleming and Jacobsson gave relatively good GFR1p determinations for the entire 2- to 5-h range (Figs. 2, 3). The GFR1p formulas also showed non-constant bias (and, to a lesser degree, variance) over the GFR range (Fig. 3), especially outside their recommended time frames. However, the Fleming and Jacobsson formulas at their best-performing time points (3 and 5 h, respectively) had an approximately constant bias and variance as a function of GFR (Fig. 3).

Subgroup analysis revealed that in children with CKD 1–3, GFR1p_3h_-Fleming scored very well, with a P10 of 92%, which was significantly better than those of all other GFR1p formulas investigated, and not significantly different from the P10 of GFR2p-JBM, which was 96% (*P* = 0.29) (Table 4). In those patients with a GFR < 30 mL/min/1.73 m^2^, much lower performances were found for all GFR1p formulas. In this subgroup, the highest P10 was 67% when the GFR1p-Fleming formula was used with blood sampling at 5 h (GFR1p_5h_-Fleming). However, the performance of GFR1p_5h_-Fleming was not significantly better than that of the GFR1p_5h_-Jacobsson which had a P10 of 44% (*P* = 0.23). In contrast, the GFR2p-JBM scored 100% for P10 (*P* < 0.0001) in the patients with GFR < 30 mL/min/1.73 m^2^ (Tables 4, 5).

Age and BSA did not seem to influence the scores of GFR1p-Fleming, whereas GFR1p-Ham&Piepsz, GFR1p-Groth&Aasted and GFR1p-Jacobsson all had better scores in the smaller children (Table 5).

## Discussion

The results of our iohexol plasma clearance study of a cohort of 96 children with CKD 1–5 shows that GFR1p measurements reached acceptable precision in patients with CKD 1–3. The best formula for single-point measured GFR in children was the GFR1p-Fleming, which showed a significantly better performance than the GFR1p-Ham&Piepsz, GFR1p-Groth&Aasted and GFR1p-Stake formulas [13, 20–22, 26] at all tested time points (Table 3; Fig. 1). GFR1p-Fleming was also significantly better than GFR1p-Jacobsson [12] when blood samplings were done after 2, 3 and 3.5 hours, whereas no significant difference was found between these formulas at 4 h (Table 4; Fig. 4). For blood sampling at 5 h, GFR1p-Jacobsson was significantly better than all other single-point formulas (Table 4; Fig. 1). Comparison with the two-point methodology showed that GFR2p-JBM, with a P10 of 97%, was significantly better (*P* < 0.001) than all single-point methods investigated in this study when all CKD stages were included in the analysis. However, an interesting finding was evident from the subgroup analysis, which showed no significant difference between the best single-point method, GFR1p_3h_-Fleming, and GFR2p-JBM in children with CKD 1–3 (Tables 4, 5). The scores for all single-point formulas were low in children with CKD 4–5, with the best P10 of 67% compared to 100% with GFR2p-JBM (*P* < 0.001) (Tables 4, 5). McMeekin and colleagues recently compared the GFR1p_3h_-Fleming with a multi-point reference method in a combined cohort of children and adults, with a total of 411 tests (247 pediatric and 164 adult tests) [19]. These authors found that 92.7% of measures [95% confidence interval (CI) 90–95%] were within 20% of the reference. This is in accordance with the results from our cohort showing a P20 of 91% (95% CI 83–95%). Our results also support the discrepancy between formulas reported by McMeekin and colleagues who found lower P20 for GFR1p_2h_-Ham&Piepsz, GFR1p_2h_-Groth&Aasted, GFR1p_3h_-Stake and GFR1p_4h_-Jacobsson compared to GFR1p_3h_-Fleming in their cohort [19].

Our study clearly demonstrates the importance of using the optimal blood sampling time points adapted to each formula. This is especially evident in the methods described by Ham&Piepsz and Groth&Aasted [11, 26], where the performance scores of all time points outside the recommended are low (Figs. 2, 3; Table 4). Furthermore, variable performance across GFR levels has to be taken into account since both these formulas scored fairly well in children with CKD 1–2, whereas the scores were unacceptably low in children with CKD 3–5 (Table 4). As the GFR1p-Ham&Piepsz formula has been a recommended single-point method in guidelines [11] and was developed from a high number (*n* = 657) of GFR measurements [26], a higher general score should be expected. Interestingly, in our study, the P10 of GFR1p-Ham&Piepsz was very high in the group of children with CKD 2, with a P10 of 96%, but only 57% in those with CKD 3, and no patient was within 10% of the reference with a GFR of < 30 mL/min/1.73 m^2^ (Table 4). A plausible explanation could be that the reference method used in the Ham & Piepsz study was not a multipoint-method, and the development of the formula was based on GFR measurements mainly in the normal range [26].

The Groth & Aasted formula was developed in a cohort with a broader distribution of GFR [16], which could explain why the single-point scores with this formula were more evenly distributed across the different CKD groups in our study (Table 4). The cohort of Groth & Aasted was, however, considerably smaller, and their five-point reference GFR had the last time point set early (2 h) [20], which probably explains the low scores in general for GFR1p-Groth&Aasted. The fairly good scores for GFR1p-Stake in children with CKD 2–3 at 3 h in contrast to the low scores for those with CKD 1 and CKD 4–5 (Fig. 2; Table 4) are probably due to the fact that the Stake-formula was developed in a cohort of 100 children mainly with CKD 2–3 and with a two-point_3h,4h_ iohexol-GFR as reference method [13].

Both the Fleming and the Jacobsson formulas have distribution volume and time-point adaption included in the respective formulas. This gives a lower vulnerability in terms of time-point variability for blood sampling, as long as the true sampling time is used in the formula. The GFR1p_3h_-Fleming scored significantly better (*P* < 0.05) than all other formulas on their recommended time points, except for GFR1p_5h_-Jacobsson-mod._._ (*P* = 0.08) (Table 3) in the cohort as a whole, and it was not significantly different from GFR2p-JBM in the subgroups CKD 1, CKD 2 and CKD 3. The subgroup analysis also showed that age and body size did not significantly influence the scores of GFR1p-Fleming. Importantly, when a child is expected to have CKD 4–5, our study shows that a single-point methodology with blood sampling up to 5 h is not recommended and that at least two blood samples should be collected (Table 5). Calculation of the eGFR [1], despite its limitations, can be helpful in making the decision to take more than one blood-sample or not, i.e. with 30 ml/min/1.73 m^2^ as the cutoff value.

Iohexol has been increasingly used as a marker for GFR measurements in recent decades. It is a nonradioactive substance, safe, inexpensive, has low inter-laboratory variation and is stable and easy to use [4, 31, 32]. Although the GFR1p-Fleming formula was originally developed using a radioactive marker in adults and children [21], our iohexol study has shown that this formula gives an accurate mGFR determination in children with CKD 1–3.These findings are of great clinical value. For the follow-up of children with cancer treated with nephrotoxic substances, as well as for children with renal and urologic diseases and mild and moderate kidney dysfunction, it is clearly beneficial to reduce the number of blood draws from two to three to a single sample. The risk of outliers is an issue in all tests, and in a single-point procedure it is necessary to redo the test if a result is surprising, whereas using a multi-point GFR procedure it is possible to remove the outlier based on examination of the elimination curve.

A limitation of this study is the lack of inulin-based gold standard analyses, but the continuous intravenous infusion and timed urine collections necessary in inulin clearance is cumbersome, and inulin is nowadays difficult to obtain. In addition, the number of patients in our study was limited to 96 children, which reduces the power of subgroup analysis. The last time point of iohexol measurement at 5 h may limit the value of the study in patients with severely reduced kidney function. However, the validity of our study is strengthened by our comparisons of a high number of blood samples at different time points and with multiple formulas.

## Conclusion

Determination of GFR in children at all ages with CKD stage 1–3 based on iohexol plasma clearance and single-point sampling at 3 h analyzed with the Fleming formula achieved the same level of performance as the two-point method. All other tested single-point formulas had a considerably lower performance. When the GFR is lower than 30 mL/min/1.73 m^2^, a procedure with at least two blood-samples is recommended for mGFR.
